# Surveillance of Antibiotic Prescribing in Intensive Care Units in Poland

**DOI:** 10.1155/2018/5670238

**Published:** 2018-08-28

**Authors:** Ewa Trejnowska, Aleksander Deptuła, Magda Tarczyńska-Słomian, Piotr Knapik, Miłosz Jankowski, Agnieszka Misiewska-Kaczur, Barbara Tamowicz, Jakub Śmiechowicz, Remigiusz Antończyk, Paul Armatowicz, Wiktor Sułkowski, Grażyna Durek

**Affiliations:** ^1^Department of Cardiac Anesthesia and Intensive Therapy, Silesian Centre for Heart Diseases, Zabrze, Poland; ^2^Medical University of Silesia, Katowice, Poland; ^3^Faculty of Natural Sciences and Technology, University of Opole, Opole, Poland; ^4^Department of Microbiology, Ludwik Rydygier Collegium Medicum, ICU, Nicolaus Copernicus University, Bydgoszcz, Poland; ^5^3rd Department of Cardiology, Silesian Centre for Heart Diseases, Zabrze, Poland; ^6^Department of Anaesthesiology and Intensive Therapy, University Hospital, Krakow, Poland; ^7^Department of Internal Medicine, Jagiellonian University Medical College, Krakow, Poland; ^8^Department of Anaesthesiology and Intensive Therapy, Silesian Hospital, Cieszyn, Poland; ^9^Department of Anaesthesiology and Intensive Therapy, Faculty of Health Sciences, Regional Hospital in Poznan, Poznan University of Medical Sciences, Poznan, Poland; ^10^Department of Anaesthesiology and Intensive Therapy, Wroclaw Medical University, Wroclaw, Poland; ^11^Department of Cardiac Surgery and Transplantology, Silesian Centre for Heart Diseases, Zabrze, Poland; ^12^Department of General and Endocrine Surgery, Medical University of Warsaw, Warsaw, Poland; ^13^Department of Anaesthesiology and Intensive Therapy, Public Hospital, Ostrów Mazowiecka, Poland; ^14^Faculty of Health Science and Physical Education, The Witelon University of Applied Sciences, Legnica, Poland

## Abstract

Antibiotic use and microbial resistance in health care-associated infections are increasing globally and causing health care problems. Intensive Care Units (ICUs) represent the heaviest antibiotic burden within hospitals, and sepsis is the second noncardiac cause of mortality in ICUs. Optimizing appropriate antibiotic treatment in the management of the critically ill in ICUs became a major challenge for intensivists. We performed a surveillance study on the antibiotic consumption in 108 Polish ICUs. We determined which classes of antibiotics were most commonly consumed and whether they affected the length of ICU stay and the size and category of the hospital. A total of 292.389 defined daily doses (DDD) and 192.167 patient-days (pd) were identified. Antibiotic consumption ranged from 620 to 3960 DDD/1000 pd. The main antibiotic classes accounted for 59.6% of the total antibiotic consumption and included carbapenems (17.8%), quinolones (14%), cephalosporins (13.7%), penicillins (11.9%), and macrolides (2.2%), respectively, whereas the other antibiotic classes accounted for the remainder (40.4%) and included antifungals (34%), imidazoles (20%), aminoglycosides (18%), glycopeptides (15%), and polymyxins (6%). The most consumed antibiotic classes in Polish ICUs were carbapenems, quinolones, and cephalosporins, respectively. There was no correlation between antibiotic consumption in DDD/1000 patient-days, mean length of ICU stay, size of the hospital, size of the ICU, or the total amount of patient-days. It is crucial that surveillance systems are in place to guide empiric antibiotic treatment and to estimate the burden of resistance. Appropriate use of antibiotics in the ICU should be an important public health care issue.

## 1. Introduction

Antibiotic use and microbial resistance in health care-associated infections are increasing globally and, in European countries such as Poland, causing health care problems [1–3]. Increasing antibiotic resistance results in increased morbidity, mortality, and cost of health care [4, 5]. This is a common and alarming problem worldwide and in Poland.

ICU represents the heaviest antibiotic burden within the hospital [6]. ICU is also recognized as a department which creates, disseminates, and amplifies antibiotic resistance, which is important from a microbiological point of view [6, 7].

Data on antimicrobial consumption in Polish ICUs are limited. Poland participated in the European Surveillance of Antimicrobial Consumption (ESAC) project, collecting data on antimicrobial consumption in ambulatory care and hospital setting. Unfortunately, the last surveillance report from 2014 contains only data on antimicrobial consumption in ambulatory care. Poland also participated in the European Point Prevalence Survey of Healthcare Associated Infections and Antimicrobial Use (EU-PPS HAI&AU) in 2011 and in 2012 [7]. Deptułą et al. [8] showed a high prevalence of health care-associated infections among patients hospitalized in both adult and pediatric Polish ICUs. They concluded that there is a need for a national infection prevention program in Poland for these groups. Based on the above findings, we performed a surveillance study on the antibiotic consumption in Polish ICUs. We aimed to assess the antibiotic prescribing practices and the annual consumption of antibiotics in adult ICUs.

## 2. Materials and Methods

Poland currently has 420 adult and 16 pediatric ICUs. All of the ICUs were invited to participate in the surveillance study, which was conducted between April 15, 2014, and June 15, 2015. Survey questionnaires were sent to all ICUs. Requested data included the number of patient-days in the ICU, the mean length of ICU stay, and the antibiotic consumption in 2014. Classification of hospitals and ICUs was obtained from the National Health Fund and included the type of hospital (district hospital, specialist hospital, university hospital, or university hospital with a hematology department), the number of hospital beds, and the number of ICU beds. Data on antibiotic consumption were calculated dividing the amount of active substance by the amount of DDD according to the WHO Collaborating Centre for Drug Statistic Methodology. The data received from the participating ICUs in 2014 were then converted and expressed in DDD/1000 patient-days.

The regional Ethical Board reviewed and approved the study protocol. The need for informed consent was waived due to the observational nature of the study.

The majority of Polish ICUs are interdisciplinary. According to the ECDC and EU-PPS HAI&AU protocol [7], the participating ICUs were divided into groups depending on the size of the hospital to which they belonged (<250 beds, 250–500 beds, and >500 beds). University hospitals with and without a hematology department were considered as separate groups.

### 2.1. Statistical Analysis

Nonparametric rank ANOVA test, Kruskal–Wallis test, post hoc tests (for multiple comparisons), chi-square test, and multipartite tables were used as appropriate for the biggest reliability of assessed relation between selected factors and antibiotic consumption in ICUs.

## 3. Results

In total, 134 adult ICUs sent their data for analysis. However, queries were sent for any unclear information. If a query remained unanswered, that particular ICU was removed from the study. Finally, data from 108 ICUs (25.7% of all adult ICUs) were included in the study. The information from each ICU was reviewed, and the data were then converted to DDD/1000 patient-days. A total of 292,389 DDD and 192,167 patient-days were included in the analysis.

**Figure 1:**
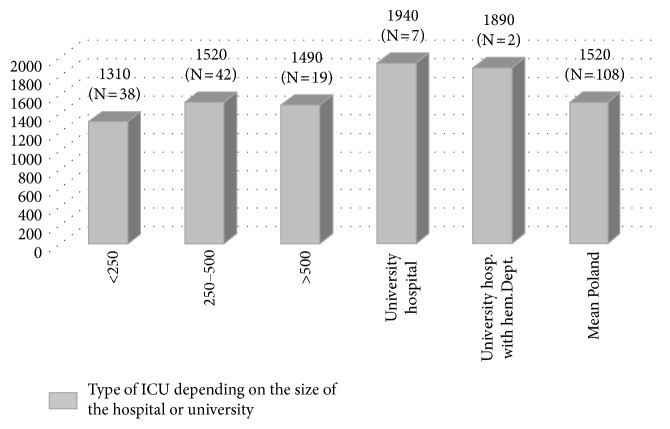
Mean antibiotic consumption in Polish ICUs (data expressed in DDD/1000 patient-days).

Antibiotic consumption varied from 620 to 3960 DDD/1000 patient-days, with a median of 1338 DDD/1000 patient-days and a mean of 1520 DDD/1000 patient-days (Figure 1). The differences in the value of DDD/1000 patient-days between various hospitals were not statistically significant (*p*=0.1287), most likely due to the high variability of results observed within the ICUs.

In hospitals with less than 250 beds, 250–500 beds, and more than 500 beds, the mean amount of DDD/1000 patient-days was 1310, 1520, and 1490, respectively. However, the highest consumption (without statistical significance) was observed in university hospitals and university hospitals with a hematology department.

In university hospitals, the mean amount of DDD/1000 patient-days was 1940, whereas in university hospitals with a hematology department, the mean amount of DDD/100 patient-days was 2% lower (Figure 1).

**Figure 2:**
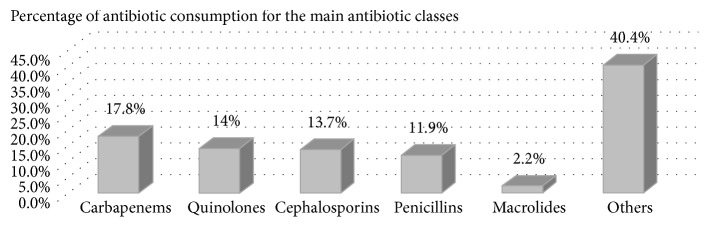
Distribution of total antibiotic consumption in Polish ICUs by main antibiotic classes.

The main antibiotic classes accounted for 59.6% of the total antibiotic consumption. Carbapenems ranged from 1% to 54% of the total antibiotics consumed with a mean of 17.8%, quinolones ranged from 1% to 44% with a mean of 14%, cephalosporins ranged from 1% to 45% with a mean of 13.7%, penicillins ranged from 1% to 58% with a mean of 11.9%, and macrolides ranged from 1% to 15% with a mean of 2.2%, whereas the other antibiotic classes accounted for the remainder (40.4% of total antibiotic consumption). The mean values for this distribution are illustrated in Figure 2.

**Figure 3:**
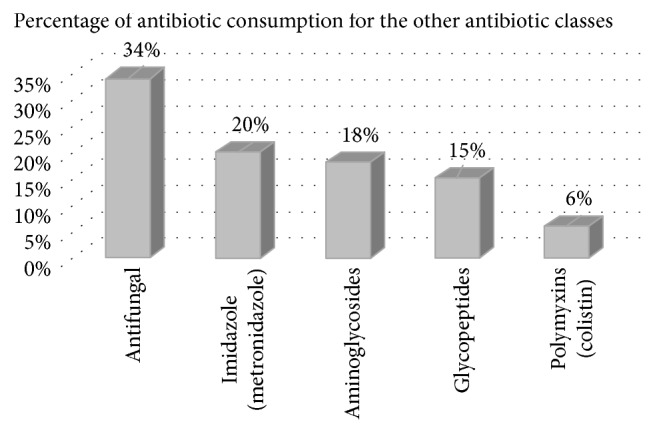
Distribution of antibiotic consumption in class “others” expressed in percentage of antibiotic consumption. Five of the most often used antibiotics in this group.

The other antibiotic classes accounted for 40.4% of the total antibiotic consumption and included antifungals (34%), imidazoles (20%), aminoglycosides (18%), glycopeptides (15%), and polymyxins (6%). Details of this comparison are shown in Figure 3.

**Figure 4:**
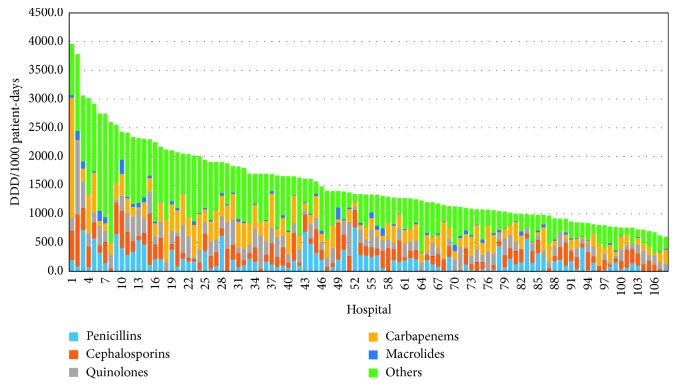
Antibiotic classes used in each of the 108 ICUs participating in the study. ICUs are presented on the *X*-axis in the order of decreasing antibiotic use.

Figure 4 displays the distribution of the various classes of antibiotics used in each ICU. Each ICU has been assigned an individual number. The main antibiotic classes are shown in different colours. The size and colour of each column correspond to the amount and type of antibiotic class used. The ICUs are presented in order of decreasing antibiotic use.

The differences in the distribution of antibiotic classes between ICUs were not statistically significant, except for macrolides and third-generation cephalosporins, which differed significantly between hospitals with more than 500 beds and university hospitals (*p*=0.037 and *p*=0.046, resp.).

The most commonly used antibiotics expressed as a percentage of total antibiotic consumption were meropenem and imipenem/cilastatin (19.6%) (meropenem 9.9% and imipenem/cilastatin 9.7%), fluconazole (13.3%), ciprofloxacin (12.2%), metronidazole (7.7%), ceftriaxone (5.8%), and vancomycin (5.4%).

The mean length of ICU stay in Poland ranged from 4.8 days to 35 days with a mean of 11.4 days. There was no correlation between mean length of ICU stay, size of the hospital, size of ICU, antibiotic consumption in DDD/1000 patient-days, and the total amount of patient-days.

## 4. Discussion

To the best of our knowledge, this is the first surveillance study investigating the antibiotic prescribing practices and the annual consumption of antibiotics in adult ICUs in Poland. 108 ICUs took part in the study, which is 25.7% of all ICUs in Poland. Our study shows that the three classes of antibiotics most consumed in Polish ICUs are the carbapenems, quinolones, and cephalosporins.

In addition, we found a high annual antibiotic prescribing rate and a substantial variation in antibiotic consumption between the ICUs. The antibiotic consumption was not dependent on the hospital size, type, or length of ICU stay.

Similar studies on the annual consumption of antibiotics in ICUs were also conducted in other European countries: in Germany, the SARI Surveillance System [9] and in Sweden, the ICU-STRAMA [10]. In comparison with these studies, in Poland, the mean annual antibiotic consumption reached 1520 DDD per 1000 patient-days, which is higher compared to Germany (1305 DDD/1000 pd in the SARI Surveillance System) [9] or Sweden (1147 DDD/1000 pd in the ICU-STRAMA report) [10]. In our study, antibiotic consumption in the ICUs ranged from 620 to 3960 DDD/1000 patient-days. This heterogeneity was also observed in a study including 29 Swedish ICUs, where antibiotic consumption ranged from 605 to 2134 DDD/1000 pd [10], and in a German SARI Surveillance System where antibiotic consumption varied from 463 to 2216 DDD/1000 pd [9]. However, the maximum annual antibiotic consumption in Sweden was similar to that found in the German study (2134 DDD/1000 pd versus 2216 DDD/1000 pd) [10], while in our study the consumption was higher, reaching 3960 DDD/1000 pd.

Our study revealed the extensive use of meropenem and imipenem/cilastatin which are used most frequently (17.8%), followed by fluconazole (13.3%), ciprofloxacin (12.2%), metronidazole (7.7%), ceftriaxone (5.8%), and vancomycin (5.4%).

According to the ECDC Surveillance Report on antimicrobial consumption during the period 2008–2012 in Europe, the most frequently consumed class of antibiotics was penicillins, followed by cephalosporin and quinolones [2]. In our study, cephalosporins were consumed 25% less often (13.7% versus 19%) and macrolides four times less often, when compared to the data from the SARI study [9]. The consumption of carbapenems in Poland was 25% higher in comparison with other countries in northern Europe [9]. The Polish Severe Sepsis Registry [11] showed that the most frequent cause of septic shock in patients admitted to the ICU was an intra-abdominal infection; hence, there is a necessity of treating Gram-negative bacterial flora [12].

In our study, the most common antifungal drug used in Polish ICUs was fluconazole, accounting for 97% of all the antifungal drugs. The reason for such high consumptions of fluconazole is multifarious. Therapeutic management in the prevention and treatment of fungal infections in Poland, as in other countries, is obliged to rely on European [13] or American guidelines [14]. However, in Polish ICUs, usage of antifungal drugs is based on the presence of risk factors for systemic fungal infections-*Candida* score [15], the respiratory culture result, or because of long-lasting antibiotic therapy. Due to the fact that it is used as prophylaxis for a fungal infection, it is easier to implement a cheaper therapy with the use of fluconazole. However, fluconazole can be successfully used in hospitals because the majority of infections are *Candida albicans* or *Candida parapsilosis* [7]. Echinocandins are left for targeted therapy or for patients in whom there are indications; for example, they have a multisite colonization of *Candida* nonalbicans [16]. The study showed for the first time how often fluconazole is used and indicates the need to increase the awareness in the field of therapy for fungal infections. This is a very high figure when compared to the 2012 ECDC surveillance on antimicrobial consumption in Europe. This surveillance has also identified 11 European countries (61%), where fluconazole alone accounted for more than 50% of the total antifungal drugs consumed. Poland seems to be a country with one of the highest consumptions of fluconazole [2]. Paying attention to such high consumptions of fluconazole is one of the greatest achievements of this study.

Excessive use of carbapenems leads to higher antimicrobial resistance which results in the use of broad-spectrum antibiotics in the ICU. This is an unsettling phenomenon which occurs in other countries in eastern and southern Europe. These observations are consistent with the data published in the annual report of the European Antimicrobial Resistance Surveillance Network (EARS-Net) [17]. The results presented in this report are based on antimicrobial resistance data from invasive isolates reported to EARS-Net by 30 European Union (EU) and European Economic Area (EEA) countries in 2017 (data referring to 2016). As in previous years, the antimicrobial resistance situation in Europe displays wide variations depending on the bacterial species, antimicrobial group, and geographical region. For several bacterial species-antimicrobial group combinations, a north-to-south gradient and a west-to-east gradient are evident in Europe. In general, lower resistance percentages were reported by countries in the north, while higher percentages were reported in the south and east of Europe. These differences are most likely related to variations in antimicrobial use, infection prevention, and control practices, and dissimilarities in diagnostic and health care utilization patterns in the countries [17].

The dynamics of antibiotic resistance are multifarious. Antibiotic usage in the animal and plant industry is a major contributor to antimicrobial resistance (AMR) [18]. Within the hospital, the ICU represents the heaviest antibiotic burden [6]. In the ICU setting itself, causes of AMR may conveniently be categorized by procedure-related, management-related, and antibiotic-related factors. Procedure-related factors include central venous catheters [18, 19], and endotracheal intubation for mechanical ventilation [16]. Management-related factors include poor adherence to infection control policy, lack of microbiological surveillance with delayed/failed recognition of resistant isolates [18, 20], patient overcrowding [21], understaffing, and implicit spread of AMR through human vectors [22], prolonged ICU length of stay [18, 23], and pre-infection with resistant organisms at the time of ICU admission [18, 23]. Antibiotic-related factors are related to the appropriateness and duration of treatment. The use of broad-spectrum antibiotics, often as a first step in the treatment of patients with suspected infections, has a documented relationship with the development of antibiotic resistance [18, 23, 24]. Similarly, the ease of access to certain antibiotic classes, either through their availability over-the-counter in certain countries (e.g., penicillins and fluoroquinolones) or through unfounded clinician concerns of suspected bacterial infection, leads to documented AMR, although causation proves difficult at an individual patient level [18].

Variability in antibiotic prescribing cannot be explained only by differences in the epidemiology of the community and hospital infections or antimicrobial resistance patterns. Socioeconomic and sociocultural factors, as well as the way in which health care is funded or reimbursed, are likely to influence antibiotic use [25, 26].

Mean length of ICU stay in our study ranged from 4.8 to 35 days with a mean of 11.4 days. This is much longer than the mean length of ICU stay in the SARI study in Germany (4.8 days) [9]. This may be due to the different severity of patients admitted to the Polish ICUs. In the Polish Severe Sepsis Registry, 4999 patients were registered during the 7-year period (2003–2009) [27]. These patients were admitted to the ICU in critical condition, and the majority of them (89%) had a dysfunction of three or more organs. The APACHE II score on admission was 26 points. In the United States, only 36% of identified patients were mechanically ventilated at some point during their ICU stay [28]. In Poland, the majority of patients (89%) admitted to the ICU were intubated [27].

One of the reasons for the severe condition of patients admitted to Polish ICUs is the lower accessibility to intensive care beds, which is three times smaller in comparison to other European countries [28]. In Western European countries, the highest number of intensive care beds for adults is in Germany—24 beds per 100 000 inhabitants [29]. In the United States, this number was 28 in 2007 [30, 31]. In Poland, the number of intensive care beds per 100 000 inhabitants is 7.1 [28]. Less available beds are associated with a delayed admission to the ICU, and as a consequence, more organ dysfunction.

This study has some important limitations. Participation in the surveillance study was voluntary, and therefore selection bias cannot be excluded. It is possible, that some ICUs encountering higher antibiotic consumption and higher resistance rates may be aware of the problem and did not wish to take part in the surveillance.

Our study describes the amount of antibiotic consumption in Polish ICUs. The wide variation in consumption between individual hospitals suggests that there is a potential for quality improvement and benchmarking. We also observed differences between our data and similar data from other countries. Results from such assessments should be distributed to both politicians and professionals in the country. It is essential to perform regular assessments of antibiotic consumption and antibiotic resistance in Polish ICUs. Updating international guidelines for appropriate antibiotic use and implementing these guidelines (e.g., including internationally agreed-upon stewardship programs) is one possibility.

Surveillance on continuous antibiotic consumption and evaluation of prescribing patterns with feedback is particularly important. Surveillance on antibiotic consumption is one of the main instruments for improving rational antibiotic use in ICUs at the regional and national level.

## 5. Conclusion

The three classes of antibiotics most consumed in Polish ICUs are the carbapenems, quinolones, and cephalosporins. The consumption of these 3 classes of antibiotics should be limited and controlled in ICUs, considering the high risk of antimicrobial resistance that involves their use.

Further research is required to determine the appropriate use of antibiotics in the ICU and should be an important safety and health care issue worldwide.

## Data Availability

The data used to support the findings of this study are available from the corresponding author upon request.
